# Implementation of blinded outcome assessment in the Effective Verruca Treatments trial (EverT) – lessons learned

**DOI:** 10.1186/s13047-016-0155-4

**Published:** 2016-07-12

**Authors:** Sarah Cockayne, Catherine Hewitt, Farina Hashmi, Kate Hicks, Michael Concannon, Caroline McIntosh, Kim Thomas, Jill Hall, Judith Watson, David Torgerson, Ian Watt

**Affiliations:** Department of Health Sciences, York Trials Unit, University of York, York, UK; School of Health Sciences, University of Salford, Salford, UK; The School of Human & Health Sciences, Division of Podiatry, University of Huddersfield, Huddersfield, UK; Discipline of Podiatry, The National University of Ireland, Galway, Republic of Ireland; Centre of Evidence Based Dermatology, University of Nottingham, Nottingham, UK; The Usher Institute of Population Health Sciences and Informatics, University of Edinburgh, Edinburgh, UK; Hull York Medical School, York, UK

**Keywords:** Blinding, Digital photographs, Outcome assessment, Randomised controlled trial

## Abstract

**Background:**

Trials using inadequate levels of blinding may report larger effect sizes than blinded studies. It has been suggested that blinded outcome assessment in open trials may in some cases be undertaken by assessments of photographs. The aim of this paper is to explore the effect of using different methods to assess the primary outcome in the EVerT (Effective Verruca Treatments) trial. It also aims to give an overview of the experiences of using digital photographs within the trial.

**Methods:**

We undertook a secondary analysis to explore the effect of using three different methods to assess the primary outcome in the EVerT trial: assessment of digital photographs by blinded healthcare professionals; blinded healthcare professional assessment at the recruiting site and patient self-report. The verruca clearance rates were calculated using the three different methods of assessment. A Cohen’s kappa measure of inter-rater agreement was used to assess the agreement between the methods. We also investigated the experiences of healthcare professionals using digital photographs within the trial.

**Results:**

Digital photographs for 189 out of 240 (79 %) patients in the trial were received for outcome assessment. Of the 189 photographs, 30 (16 %) were uninterpretable. The overall verruca clearance rates were 21 % (43/202,) using the unblinded patient self-reported outcome, 6 % (9/159,) using blinded assessment of digital photographs and 14 % (30/210,) using blinded outcome assessment at the site.

**Conclusions:**

Despite differences in the clearance rates found using different methods of outcome assessment, this did not change the original conclusion of the trial, that there is no evidence of a difference in effectiveness between cryotherapy and salicylic acid. Future trials using digital photographs should consider individual training needs at sites and have a backup method of assessment agreed *a priori*.

**Trial registration:**

ISRCTN Registry ISRCTN18994246

## Background

Blinding is widely used in randomised controlled trials to minimise the possibility of introducing bias as a result of those involved in the study being aware of which treatment has been received [1]. There are several potential issues if patients, investigators and outcome assessors are aware of the treatment allocation. First, patients who know they have received a new treatment may hold either favourable expectations or increased apprehension, whilst those receiving the standard treatment may feel deprived or relieved. Such knowledge may affect psychological or physical responses and could influence the patient’s cooperation in the trial for example attendance for evaluation [2]. Second, investigators who are aware that patients are receiving a novel intervention may follow their progress more closely than those on standard treatment. Alternatively they may transfer their opinion either for or against a treatment to the patient which may affect the patient’s attitude [3]. Finally, for outcome assessors there is the potential to report more favourable outcomes for those in the novel intervention group if they believe it to be a superior treatment. However, this is more likely to be an issue where more subjective outcomes such as pain scores are being assessed rather than objective outcomes such as death.

Trials which have not used appropriate levels of blinding have been shown to report larger effect sizes than blinded studies [4]. It has been suggested that blinded outcome assessment in open trials may in some cases be undertaken by assessment of photographs [5] and digital photographs have been used previously in randomised controlled trials to assess outcome [6, 7]. Digital photographs are a useful way of capturing and demonstrating global changes in lesions as well as providing objectivity and reproducibility. It may be possible to use computer algorithms to identify the lesion and analyse its size and shape, as well as identify any irregularity of colour and segmentation [8]. There are several advantages to using digital photographs for outcome assessment. First, it eliminates the verbal and non-verbal clues about group allocation and allows the same assessors to evaluate the outcome for all participants in a study. Second, it assists with demonstrating the transparency of the data. Finally, it may assist with centrally monitoring the study, for example confirmation of the existence of patients which may help identify fraudulent behaviour or validate compliance with the trial protocol over the number of treatment visits. Used in conjunction with telecommunications, digital imaging can extend the reach of patient participation in studies. However, a number of disadvantages of using digital photographs also exist, including the purchase cost of equipment, although this has reduced in recent years; the time to take and process photographs; and the need to train staff. Furthermore, additional time is required to handle the photographs at the study coordinating centre.

The EVerT (Effective Verruca Treatments) trial compared the use of cryotherapy delivered by a healthcare professional to self-administered salicylic acid. As such, it was not possible to blind the patients or the healthcare professionals to their treatment allocation. In order to ensure adequate blinding outcome assessments were undertaken in two ways. Firstly a digital photograph of the verruca(e) was taken. As far as we are aware, this is the first study to undertake this type of assessment in such a trial. Second a healthcare professional at the site, who was not involved in treating the participants, assessed the patient to determine whether their verruca(e) was/were still present. It was agreed *a priori* to use the digital photos as the means of assessment, and assessment at the site would only be used if this was uninterpretable. The aim of this paper is to undertake a secondary analysis to explore the effect of using different methods to assess the primary outcome in the EVerT trial. It also aims to give an overview of the experiences of using digital photographs in the trial in order to inform future dermatology trials.

## Methods

### The EVerT trial

The EVerT trial was a multicentre, two arm randomised controlled open trial evaluating the clinical and cost effectiveness of salicylic acid and cryotherapy for the treatment of verrucae. The study was approved by Trent Multicentre Research Ethics Committee (MREC reference 04/mre04/59), Galway Research Ethics Committee, local research ethics committees, Medicines and Healthcare products Regulatory Agency, Irish Medicines Board and local Research and Development Trusts. All patients provided written informed consent prior to being enrolled in the study. Detailed methods [9, 10] and the main trial results have been published elsewhere [11]. In brief, 240 patients were recruited from University podiatry schools, NHS podiatry clinics and primary care. Patients were eligible for the study if they were over 12 years of age and had at least one verruca which was suitable for treatment with both trial treatments. Patients were randomly allocated to receive cryotherapy using liquid nitrogen or self-treatment with 50 % salicylic acid. The primary outcome was complete clearance of all verrucae at 12 weeks after randomisation as observed on digital photographs by blinded podiatrists and by blinded assessment at the recruiting site by podiatrists, General Practitioners and Practice Nurses. Patient self-reported clearance rates at 12 weeks were also obtained via postal questionnaire.

### Digital photographs and outcome assessment methods

Study sites were requested to take digital photographs at baseline and at the 12 week outcome assessment visit. The photographs were taken using either a Nikon Coolpix L11 digital camera provided by the trial, or the Healthcare Professional’s (HCP’s) own camera if it was a similar specification. Sites were given written guidelines on how to use the camera. Digital photographs received by the coordinating centre were reviewed and further advice given by telephone in cases where the quality of the photographs received would make it difficult to interpret. The photographs were assessed by two assessors who were blind to treatment allocation. They independently assessed the photographs for each participant to determine whether the verrucae had cleared. Any discrepancies were referred to a third assessor. The assessors were asked to record if the photograph was uninterpretable and the reasons why and record if they knew for certain which treatment the patient received. Patient self-reported clearance rates at 12 weeks were also obtained via postal questionnaire. Participants who did not attend their 12 week outcome assessment visit were asked to take a digital photograph of their foot and email it to the coordinating centre.

### Statistical analysis

The number of uninterpretable photographs by centre were summarised descriptively. The available outcome assessment data were summarised descriptively along with the baseline data and effectiveness of the blinding.

The verruca clearance rates were reported using the three different methods of assessment and a Cohen’s kappa measure of inter-rater agreement was used to assess the agreement between the methods of assessment. We fitted a logistic regression model with verruca clearance (yes/no) as the primary outcome and treatment, age, type of verruca and previous treatment as covariates. Three analyses were undertaken, one using the digital photograph reported outcome at 12 weeks, one using the blinded outcome assessment at the site at 12 weeks and one using patient’s self-reported outcome at 12 weeks. The results of these analyses were then reported graphically.

All analyses were conducted on an intention-to-treat basis, including all patients in the groups to which they were randomised. All analyses were conducted using Stata version 13.0 (Texas, USA) using two sided significance tests at the 5 % significance level.

## Results

### Secondary analysis to explore the effect of using different methods of outcome assessment

**Fig. 1:**
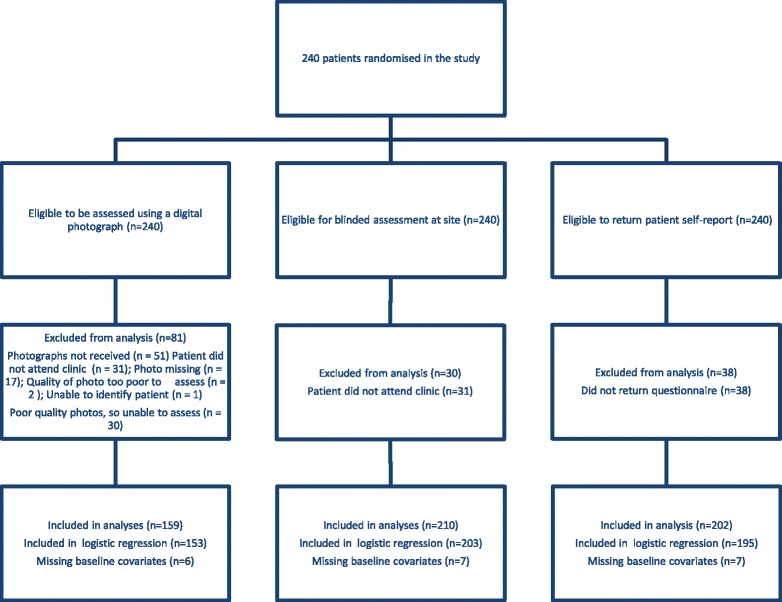
Flow of patients through the trial

Figure 1 shows the flow of patients through the trial. In total 240 eligible patients were recruited to the study. Digital photographs were taken at the 12 week outcome assessment point and the coordinating centre received photographs for 189/240 (79 %) patients. The coordinating centre did not successfully receive photographs for 51 patients and the reasons for the missing data were as follows: patient did not attend their outcome assessment and so no photograph was taken (*n* = 31); photographs were missing (*n* = 17); unclear photographs not given to the assessor (*n* = 2); and not possible to identify which patient the photograph related to (*n* = 1). The number of uninterpretable photographs by centre and type of healthcare professional is documented in Table 1. Of the 189 photographs given to the blinded assessors, 30 (16 %) were deemed by the assessors to be of insufficient quality to allow an assessment to be undertaken (Table 1). In the majority of these cases (*n* = 27) the photograph could not be interpreted because it was not in focus. The digital photograph assessors reported 16 cases where they were aware of the treatment the patient received. In all cases they reported that the patient had received treatment with salicylic acid, an assumption which on review of the participant’s group allocation was found to be correct in 14 of the 16 cases. For 210 of the 240 (88 %) patients in the study had blinded outcome assessments at the site. Patient self-reported clearance of verrucae via the 12-week postal questionnaire was available for 202/240 (84 %) patients. A summary of the available outcome assessment data for patients in the study is presented in Table 2.

**Table 1:** Number of uninterpretable photographs by centre and type of healthcare professional

Centre ID number	Type of healthcare professional	Number of photographs	Number of uninterpretable photographs^a^	Take digital photos as part of their routine work
1	Podiatrist^b^	48	0	Yes
2	GP	1	0	No
3	Practice nurse	4	2	No
4	Podiatrist	9	1	Yes
5	Podiatrist^b^	33	4	Yes
6	GP	12	0	No
7	Podiatrist	11	0	Yes
8	Practice nurse	5	1	No
9	Podiatrist	8	2	No
10	Podiatrist	33	16	No
11	Practice nurse	13	4	No
12	Practice nurse	3	0	No
13	GP	9	0	No
Total		189	30	

^a^ Photograph uninterpretable due to being unclear (*n* = 27); insufficient detail (*n* = 4); lesion obstructed by identifier card (*n* = 1); other reason (*n* = 1). More than one category could be checked so the total for all categories totals more than 30

^b^ HCP used their own camera; all other sites used the camera provided by the trial

**Table 2:** Summary of available outcome assessment data

Data available	Number of patients
	*N* = 240
Photograph, blinded assessment at site and patient self-report	142
Blinded assessment at site and patient self-report (no photograph)	41
Photograph and blinded assessment at site (no patient self-report)	17
Photograph and patient self-report (no blinded assessment at site)	0
Only had photograph (no blinded assessment at site and no patient self-report)	0
Only had blinded assessment at site (no photograph and no patient self-report)	10
Only had patient self-report (no photograph and no blinded assessment at site)	19
Missing data (no photograph, no blinded assessment at site or patient self-report)	11

**Fig. 2:**
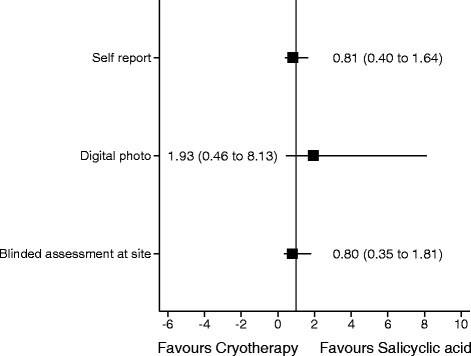
Forest plot of assessment method comparing salicylic acid and cryotherapy for treatment of verrucaeᅟ

Table 3 presents the descriptive statistics for the baseline characteristics of patients and their presenting verrucae between the three types of outcome assessment. In general the groups were balanced at baseline. The clearance rates of verrucae by assessment method are reported in Table 4. The Cohen’s kappa measure of agreement between the three methods of assessment was estimated as 0.61 (*p* < 0.001). This indicates a good level of agreement between the three methods. Results of the logistic regression model of verruca gone status were as follows: assessment by photograph OR 1.93 (95 % CI 0.46 to 8.13) *p* = 0.371: blinded assessment at site: OR 0.80 (95 % CI 0.35 to 1.81) *p* = 0.593 and patient self-report OR 0.81 (0.40 to 1.64) *p* = 0.553. None of the assessment methods resulted in a statistical significant result. These results are reported graphically in Fig. 2.

**Table 3:** Baseline characteristics of participants according to type of outcome assessment

	Photograph	Blinded assessment at site	Patient self-report
	(*N* = 159)	(*N* = 210)	(*N* = 202)
Age (years)			
N, Mean (SD)	158, 30.8 (16.6)	209, 30.7 (16.4)	200, 30.9 (16.7)
Median (min, max)	24.5 (12.0, 75.3)	24.2 (12.0, 75.3)	24.3 (12.0, 75.3)
Missing	1	1	2
Gender			
Female (%)	109 (69.0)	140 (67.0)	136 (68.0)
Male (%)	49 (31.0)	69 (33.0)	64 (32.0)
Missing	1	1	2
Type of verrucae			
Mosaic, n (%)	36 (23.4)	42 (20.6)	46 (23.4)
Non-mosaic, n (%)	118 (76.6)	162 (79.4)	151 (76.7)
Missing	4	6	5
Duration of verrucae (months)			
N, Mean (SD)	149, 29.5 (26.7)	199, 26.9 (25.2)	192, 26.9 (25.5)
Median (min, max)	24 (1, 144)	18 (1, 144)	19.1 (1, 144)
Missing	10	11	10
Number of verrucae at baseline			
N, Mean (SD)	152, 4.0 (5.9)	201, 3.8 (5.5)	194, 3.9 (5.5)
Median (min, max)	2 (1, 55)	2 (1, 55)	2 (1, 55)
Missing	7	9	8
Previous treatment			
Yes, n (%)	130 (82.3)	163 (78.0)	161 (80.5)
No, n (%)	28 (17.7)	46 (22.0)	39 (19.5)
Missing	1	1	2
Type of previous treatment^a^			
Self-treatment, n (%)	113 (86.9)	144 (88.3)	141 (87.6)
Podiatrist/chiropodist, n (%)	41 (31.5)	47 (28.8)	43 (26.7)
GP, n (%)	56 (43.1)	65 (40.0)	64 (39.8)
Trial investigating verruca treatments, n (%)	2 (1.5)	2 (1.2)	2 (1.2)
Other, n (%)	12 (9.2)	14 (8.6)	14 (8.7)

^a^ More than one category could be checked so the total for all categories may total more than 100 %

**Table 4:** Verruca clearance rates by assessment method

Clearance	Cryotherapy Number (%)	Salicylic acid Number (%)	Total Number (%)
Digital photograph			
Gone	3 (3.8)	6 (7.6)	9 (5.7)
Not gone	77 (96.2)	73 (92.4)	150 (94.3)
Total	80 (100.0)	79 (100.0)	159 (100.0)
Blinded assessment at site			
Gone	17 (16.5)	13 (12.2)	30 (14.3)
Not gone	86 (83.5)	94 (87.9)	180 (85.7)
Total	103 (100.0)	107 (100.0)	210 (100.0)
Patient self-report			
Gone	21 (21.9)	22 (20.8)	43 (21.3)
Not gone	75 (78.1)	84 (79.2)	159 (78.7)
Total	96 (100.0)	106 (100.0)	202 (100.0)

The overall verruca clearance rate reported using the patient self-reported outcome data was higher than the rates using both blinded digital photograph assessment and blinded healthcare professional assessment at the site. Although there was little or no evidence of difference in clearance rates using any of the methods of assessment, there was a change in the direction of the effect for the blinded digital photograph assessment compared to the other forms of assessment. Assessment using digital photographs reported slightly higher cure rates with salicylic acid compared to cryotherapy and blinded outcome assessment at the site and patient self-reported outcomes reported higher cure rates with cryotherapy compared to salicylic acid.

### Our experiences of using digital photographs

All the patients in this study consented to have photographs taken. Healthcare professionals who either took photographs as part of their routine clinical practice or had an interest in photography had few difficulties with taking and processing the photographs. However, other HCPs found the process challenging, time consuming and often took out of focus photos. This was despite attempts to improve their quality e.g. by reviewing photos at the site whilst the patient was still present and taking multiple photographs. One site which did not routinely take photos as part of their routine work, experienced such difficulties in taking photographs with the camera provided to them by the trial, that they requested assistance from the trust’s medical photographer. Whilst they were willing to take the photographs, this was not practically possible due to their considerable distance from the podiatry department. In 13 cases where patients did not attend their outcome assessment, the coordinating centre wrote to them asking if they would send a digital photograph. One patient complied with this request.

Anecdotally, assessors reviewing the digital photographs reported several reasons why they were unable to interpret them. In some cases the photograph was taken too far away to allow close inspection. However, if the zoom facility was used it sometimes resulted in the image being blurred or distorted. Problems with lighting resulting in glare or shadows also made assessment difficult. Reviewers found it helpful to have more than one concurrent photograph as it either increased the chances of obtaining a better quality photograph or helped in cases where the lesion extended around the foot or where both feet were affected. However, if the baseline photograph was unclear, assessing the follow up photograph for any change was problematic. Assessors found it frustrating not to be able to review the patient in person, as they said it would have helped in their assessment. Although not only an issue for assessment using digital photographs, in some cases it was possible to see that the patient had been treated with salicylic acid due to the circular shaped macerated area surrounding the verrucae, which resulted from treating with the salicylic acid.

Other trials managed by the York Trials Unit have reported additional issues with handling digital photographs. Although it was not an issue in this study, some NHS Trusts only allow medical imaging staff to take photographs of patients. Whilst this improves the quality of the photographs received, it increases costs and the amount of time spent at the clinic for patients. Other NHS Trusts do not allow non-NHS software to be uploaded onto their computers. This resulted in memory cards having to be sent back to the coordinating site.

## Discussion

This study has demonstrated that it is possible to use digital photographs to assess verruca clearance rates. However some potential problems are highlighted that researchers should be aware of. There was a variation between sites as to the quality of the photographs taken and so individualised training needs should be assessed. It also highlights the need to have a back-up option in place in case the image fails. Using a combination of outcome assessment methods allowed us to minimise the amount of missing data in this study. However, the type and priority of data to be used for analysis should be agreed *a priori.*

As we anticipated the verruca clearance rates were highest when they were self-reported by participants. This could be due partly to the fact that the patients were unblinded to the treatment they received. Some patients allocated to cryotherapy may have reported higher cure rates because they believed the treatment must have been effective as the treatment was painful. Similarly the podiatrists undertaking the blinded outcome assessment both at site and on the photos may have over or under reported clearance rates in those cases where they were able to correctly identify the patient had been treated with salicylic acid. Alternatively it could be due to the healthcare professionals using different criteria on which to define clearance. Healthcare professionals were asked to review whether there was “restoration of normal skin upon close inspection”, whilst patients were asked if their verruca(e) had gone or not and were not given any criteria on which to make this judgement. It may have been possible that patients, who reported their verruca as being unsightly and/or painful, may have used these criteria on which to base whether or not their verruca had been cured, if these signs and symptoms were reduced after treatment. It has been suggested that patients may be able to detect changes in their lesions if they are given the opportunity to review the original lesion on a photograph [12]. The overall cure rate reported by patients may therefore have been lower if this type of assessment had been undertaken. Whilst the clearance rates as assessed by digital photograph or assessment at the site varied considerably this is most likely due to the small numbers involved with the low cure rate. In this case the largest effect size was not seen in the unblinded assessment but in the blinded assessment at the site. Whilst there was a change in the direction of effect when using digital photographs compared to assessment at the site or patient self-reported outcomes, none of the assessment methods resulted in a statistically significant result. The conclusion of the study therefore did not change with the type of assessment used, i.e. there was no evidence of a difference in effectiveness between the treatments. However care should be taken in interpreting these results due to the very low overall cure rates.

Manual tracking of digital photographs by the trial coordinating centre was a time consuming process. However, there are now automated systems, whereby photographs are uploaded directly which would overcome this problem. Whilst it might have been possible to obtain higher quality pictures if using a higher specification of camera there are of course cost and training implications. Whilst technology has improved the potential for taking poor quality photos still remains a possibility. In this study staff often took multiple photos. Whilst these photos were reviewed at the point of taking them, some sites reported that the photograph looked in focus on the LCD screen but when uploaded onto the computer was then found to be out of focus. In future trials, attention needs to be paid to digital image reliability, reproducibility, security and usefulness to ensure issues surrounding authentication, manipulation, audit trail verification and data compression are considered [13].

## Conclusions

In conclusion, although there was a difference in the direction of the effect for verruca clearance rates using data from blinded digital photographs, blinded assessment at the site and unblinded patient self-report, none of the results reached statistical significance. The original conclusion drawn from the study i.e. that there is no evidence of a difference in effectiveness between cryotherapy using liquid nitrogen and daily self-treatment with 50 % salicylic acid is not changed. In future trials we would recommend that if digital photographs are to be used as a means of outcome assessment, then training needs at individual sites should be assessed and more than one photograph should be taken at each time point and an automated process of handling photographs should be used. We would suggest having a backup method of assessment but it should be agreed *a priori* which data should be used.

## Abbreviations

EverT, Effective Verruca Treatment; HCP, healthcare professional; MREC, Multicentre Research Ethics Committee.
